# Cellulose synthase interactive1- and microtubule-dependent cell wall architecture is required for acid growth in Arabidopsis hypocotyls

**DOI:** 10.1093/jxb/eraa063

**Published:** 2020-02-04

**Authors:** Xiaoran Xin, Lei Lei, Yunzhen Zheng, Tian Zhang, Sai Venkatesh Pingali, Hugh O’Neill, Daniel J Cosgrove, Shundai Li, Ying Gu

**Affiliations:** 1 Department of Biochemistry and Molecular Biology, Pennsylvania State University, University Park, PA, USA; 2 Department of Biology, Pennsylvania State University, University Park, PA, USA; 3 Biology and Soft Matter Division, Oak Ridge National Laboratory, Oak Ridge, TN, USA; 4 INRAE-University Clermont Auvergne, France

**Keywords:** Acid growth, atomic force microscopy, axial elongation, cell wall, crossed-polylamellate walls, electron microscopy, microtubules

## Abstract

Auxin-induced cell elongation relies in part on the acidification of the cell wall, a process known as acid growth that presumably triggers expansin-mediated wall loosening via altered interactions between cellulose microfibrils. Cellulose microfibrils are a major determinant for anisotropic growth and they provide the scaffold for cell wall assembly. Little is known about how acid growth depends on cell wall architecture. To explore the relationship between acid growth-mediated cell elongation and plant cell wall architecture, two mutants (*jia1-1* and *csi1-3*) that are defective in cellulose biosynthesis and cellulose microfibril organization were analyzed. The study revealed that cell elongation is dependent on CSI1-mediated cell wall architecture but not on the overall crystalline cellulose content. We observed a correlation between loss of crossed-polylamellate walls and loss of auxin- and fusicoccin-induced cell growth in *csi1-3*. Furthermore, induced loss of crossed-polylamellate walls via disruption of cortical microtubules mimics the effect of *csi1* in acid growth. We hypothesize that CSI1- and microtubule-dependent crossed-polylamellate walls are required for acid growth in Arabidopsis hypocotyls.

## Teaser

We observed a correlation between acid growth and crossed-polylamellate cell walls which was dependent on CSI1 and microtubules. This is significant for re-evaluating current models of cell morphogenesis.

## Introduction

Anisotropic cell expansion is a characteristic feature of plant cells. Cell expansion results from the interplay between uniform turgor pressure and a yielding cell wall. The control of the direction of cell elongation resides in the anisotropic cell wall structure. Cellulose is considered the major load-bearing polymer in the primary cell wall. Therefore, its role in regulating directional cell expansion has been extensively studied (Gertel and Green, 1977; Refregier *et al*., 2004; Derbyshire *et al.*, 2007; Park and Cosgrove, 2015; Videcoq *et al.*, 2017). A widely cited hypothesis states that the orientation of cellulose microfibrils is perpendicular to the axis of maximum cell elongation. In other words, transversely oriented cellulose microfibrils in the cell wall favor expansion in the long axis and restrict lateral expansion, an arrangement likened to ‘hoops around a barrel’ (Green, 1960, 1962). However, transverse orientation of cellulose microfibrils is not always correlated with a rapid cell elongation rate. Axially aligned cellulose microfibrils were observed at the innermost cell wall in elongating parenchyma cells (Itoh, 1975; Itoh and Shimaji, 1976). The inhibition of elongation in *procuste*, a cellulose synthase mutant of Arabidopsis, was accompanied by normal deposition of transverse orientation of nascent cellulose microfibrils in hypocotyl epidermal cell walls (MacKinnon *et al.*, 2006). Similarly, cellulose microfibrils in the innermost cell wall layer were transversely oriented in the elongation zone of *rsw4* and *rsw7* roots in which cell elongation was drastically reduced (Wiedemeier *et al.*, 2002). The conflicting data may partly be explained by the differences in regulation in aerial versus root tissues. Moreover, early studies are limited by failure to observe dynamic reorganization of cellulose microfibrils as inferred by visualization of cellulose synthase (CESA) trajectories (Chan *et al.*, 2010). To add another layer of complexity, the alignment of cellulose microfibrils at the inner and outer periclinal walls of epidermal cells differs significantly (see Supplementary Fig. S1 at *JXB* online). Recent discussions have been centered on which cell wall feature is a better predictor of growth anisotropy. Because the outer epidermis has a nearly net isotropic orientation of cellulose, two studies have favored the role of inner periclinal walls of the epidermis in the regulation of growth anisotropy (Chan *et al.*, 2011; Crowell *et al.*, 2011).

However, the outer epidermal cell walls bear most of the stress exerted by the expanding internal tissues and represent a growth-limiting sheath for multicellular systems (Green, 1980; Kutschera, 2008; Savaldi-Goldstein and Chory, 2008). The outer periclinal epidermal walls (~1 μm) of Arabidopsis hypocotyl are much thicker than the inner periclinal walls (Derbyshire *et al.*, 2007). The inner periclinal walls (~50 nm) are estimated to accommodate at most 1–2 microfibrillar lamellae. In the shoot apical meristem, the outer periclinal epidermal wall is approximately seven times thicker than the inner periclinal wall (Kierzkowski *et al.*, 2012). The outer periclinal epidermal walls in onion epidermal cell have ~100 lamellae with different cellulose orientations between each successive lamella (Zhang *et al.*, 2016). Consistent with the role of the outer periclinal epidermal walls in limiting elongation of the intact organ, studies have shown that outer epidermal-specific expression of the brassinosteroid receptor or a brassinosteroid biosynthetic enzyme in null mutants was sufficient to rescue their dwarf phenotypes in Arabidopsis, indicating that molecular components involved in perception of external cues and in transducing into mechanical sensing/wall modification reside in the outer epidermis (Savaldi-Goldstein and Chory, 2008). It remains unclear whether and how the crossed-polylamellate outer epidermal walls regulate cell elongation. It has been postulated that cell elongation is determined by the direction of net alignment among cellulose microfibrils in many cells of a tissue or organ (Baskin, 2005).

Multiple hypotheses have been proposed to explain how the crossed-polylamellate outer epidermal walls are formed. The observations that microfibril orientation changes from transverse to longitudinal from the most recently deposited cell wall to the outermost cell wall of *Nitella* suggest that a passive re-orientation of cellulose microfibrils to the axial direction occurs during growth (Green, 1960; Gertel and Green, 1977). As cell wall matrix polysaccharides are continuously deposited during growth, they may contribute to passive re-orientation of microfibrils (Gertel and Green, 1977; Preston, 1982). Alternatively, the anisotropic orientation of microfibril deposition may involve guidance by microtubules (MTs) (Chan *et al.*, 2010). The latter concept is supported by the observation that the rotation of the CESA trajectories was abolished by reagents disrupting MTs, accompanying the loss of the crossed-polylamellate wall in Arabidopsis hypocotyls (Chan *et al.*, 2010).

The role of MTs in guidance of cellulose microfibril alignment is probably not conserved in all cell types, but in general there is good agreement between the orientation of cellulose microfibrils, and the underlying cortical MTs in cells undergo rapid cell elongation (Heath, 1974; Hepler and Palevitz, 1974). Supporting the MT–microfibril alignment hypothesis, cellulose synthase complexes (CSCs) have been visualized as diffraction-limited particles that move along the underlying cortical MTs (Paredez *et al.*, 2006). Concurrent rotations have been observed at the outer epidermal cell wall of the hypocotyl for both MTs and CSC trajectories (Chan *et al.*, 2007). CELLULOSE SYNTHASE INTERACTIVE1 (CSI1) is a linker protein that mediates the interaction between CSCs and MTs (Gu *et al.*, 2010; Bringmann *et al.*, 2012; Li *et al.*, 2012); it does so by interacting directly with both CSCs and MTs (Li *et al.*, 2012). The CSC trajectories were uncoupled from the underlying cortical MTs in a *csi1* null mutant, supporting its essential role for co-alignment between CSC trajectories and MTs. As the *CSI1* mutation uncouples CSC trajectories from MTs, it serves as a tool to re-evaluate the importance of orientation of cellulose microfibrils during rapid cell elongation. An additional useful tool includes *jiaoyao1-1* (*jia1-1*) that bears a missense mutation in KORRIGAN, a membrane-associated endo-β-1,4 glucanase, which results in reduced crystalline cellulose content and decreased cellulose microfibril organization (Lei *et al.*, 2014).

In this study, the growth defects in *jia1* and *csi1* mutants were characterized in detail. The results suggest that the alignment of the most recently deposited cellulose microfibrils in the outer periclinal wall is not sufficient to explain anisotropic cell elongation in both mutants. A correlation between loss of crossed-polylamellate wall and loss of auxin- and fusicoccin (FC)-induced cell elongation was revealed.

## Materials and methods

### Plant materials and growth conditions

All seeds were surface-sterilized with 30% (v/v) bleach for 15 min, thoroughly washed with autoclaved double-distilled H_2_O (ddH_2_O), and stored at 4 °C for a minimum of 3 d. Dark-grown seedlings were grown on vertical half-strength Murashige and Skoog (MS) plates without sucrose at 21 °C in the dark. Light-grown seedlings were grown on vertical half-strength MS plates with 1% sucrose at 21 °C on a 16 h light/8 h dark cycle.

### Live-cell imaging and analysis

Images were obtained from epidermal cells of 3-day-old etiolated seedlings 0.5–2 mm below the apical hook unless indicated otherwise. Imaging was performed on a Yokogawa CSUX1 spinning-disk system as previously described (Li *et al.*, 2012). Image analysis was performed using Metamorph and ImageJ software. The average trajectory orientation was analyzed using FibrilTool, an ImageJ plug-in to quantify fibrillar structures (Boudaoud *et al.*, 2014).

### Atomic force microscopy

Around 100 seedlings of the wild type (WT; Col-0) and *csi1-3* mutant were grown on vertical half-strength MS plates without sucrose at 21 °C in the dark. Hypocotyls were ground in liquid nitrogen and rinsed with 20 mM HEPES buffer, pH 7.0, and 0.1% Tween-20 until the filtrate was clear. One droplet of well-resuspended hypocotyl walls was added onto a clean glass slide, and excess buffer was evaporated but the sample still remained visibly moist. To remove any loosely bound cell wall fragments, the wall samples were rinsed and rehydrated with 20 mM HEPES before scanning. A light microscope was used to identify the unique rectangular shape of epidermal cell wall fragments from the hypocotyls. The cell angle information was obtained during image acquisition. The atomic force microscopy (AFM) imaging was performed as previously described (Lei *et al.*, 2014). For each AFM image, the orientations of cellulose microfibrils in the most recently deposited layer were determined by measuring the cellulose microfibril angles with respect to the long axes of epidermal cells. The microfibril angles were measured using FibrilTool, an ImageJ plug-in to quantify fibrillar structures (Boudaoud *et al.*, 2014). Three regions of interest (ROIs) were selected from each AFM image for angle measurement. The microfibril angles were quantified from 28 AFM images from 13 cells for the WT and 16 AFM images from seven cells for *csi1-3*.

### Transmission electron microscopy

The apical portions of hypocotyl segments (cut ~0.5–2 mm below the apical hook) were obtained from 3-day-old dark-grown WT (Col-0), *csi1-3*, and *jia1-1* seedlings. Samples were fixed in 50 mM phosphate buffer (pH 7.0) containing 2.5% glutaraldehyde and 0.1% Triton X-100 for 3 h at room temperature. The residual glutaraldehyde was removed by washing the samples with 50 mM phosphate buffer three times for 5 min each. Samples were then immersed in DMSO for 16 h to cause cell wall swelling and washed with ddH_2_O, followed by dehydration with an ethanol series (25, 50, 60, 70, 80, 90, 100%; 10 min each); 100% EM grade ethanol three times for 10 min; and 100% acetone three times for 10 min. The dehydrated samples were infiltrated with acetone:Eponate 12 resin (2:1, 1:1, 1:2, >6 h each) and pure Eponate 12 (three times for >6 h). Samples were then embedded in a mold, in which the hypocotyl hook was positioned on the top of the mold. After incubating at 70 °C overnight, ultrathin sections (~70 nm) were cut at room temperature using an ultra-microtome (Leica EM UC6) equipped with a diamond knife and collected with gold grids.

The thin sections on the grids were incubated with 1% periodic acid in deionized water for 30 min and washed with deionized water three times before being incubated with freshly prepared 0.2% thiocarbohydrazide in 20% acetic acid for 24 h. Then the sections were washed sequentially with 10, 5, and 2% acetic acid, and deionized water. The samples were stained with 1% silver proteinate in deionized water for 30 min, followed by washing. The sections were stained with 0.46% lead citrate in deionized water for 12 min, then washed by 25 mM NaOH, 12.5 mM NaOH, and deionized water for 1 min each. Images were collected using a transmission electron microscope (FEI Tecnai TEM) under the 120 kV accelerating voltage conditions.

### Environmental scanning electron microscopy

Etiolated seedlings were gently attached to sample holders with a thin layer of carbon tape. Images were acquired using a Quanta 250 environmental scanning electron microscope (Thermo Scientific) in the high vacuum mode. The measurement of cell length was performed using ImageJ software.

### Auxin-induced cell elongation

The shoot apexes and roots of 3-day-old Arabidopsis etiolated seedlings were removed using a razor blade. The hypocotyl segments were placed in depletion medium (10 mM KCl, 1 mM MES, pH 6.0). Then the hypocotyl segments were transferred to new depletion medium supplemented with 1 μM indole-3-acetic acid (IAA) or 0.02% ethanol solvent control. Images of hypocotyl segments were acquired at the beginning of incubation and after 150 min of incubation (Fendrych *et al.*, 2016).

### Fusicoccin-induced cell elongation

The shoot apexes and roots of 3-day-old Arabidopsis etiolated seedlings were removed using a razor blade. The hypocotyl segments were placed in depletion medium (10 mM KCl, 1 mM MES, pH 6.0). Then the hypocotyl segments were transferred to new depletion medium supplemented with 2 μM FC or 0.02% ethanol solvent control. Images of hypocotyl segments were acquired at the beginning of incubation and after 150 min of incubation.

### Creep assay

The 3-day-old Arabidopsis etiolated seedlings were collected, stored at –80 °C, and thawed at room temperature prior to experiments. An entire seedling was clamped under a constant force of 2.5 g, and initial hypocotyl length was recorded. The clamped seedling was incubated in 20 mM HEPES buffer (pH 6.8) for 20 min. Then the incubation buffer was replaced with 20 mM sodium acetate buffer (pH 4.5). The wall extension was recorded every 30 s for 75 min (Cosgrove, 1998; Durachko and Cosgrove, 2009). The relative extension rate was normalized to initial sample length. For each genotype, creep responses of eight individual seedlings were recorded. One out of three reproducible independent experiments were used in the analysis.

For enzyme-induced creep, leaf petioles from the fifth to eighth leaves from 3-week-old plants were harvested, stored at –80 °C, and thawed prior to experiments. The petioles were abraded by rubbing the surface 10 times with carborundum slurry to facilitate buffer and protein penetration, and then washed with distilled water. The samples were pressed under a weight for 5 min to remove residual cell fluids and to flatten the wall sample, as described previously (Park and Cosgrove, 2012*a*, *b*). The pressed wall specimens were heat inactivated in boiling water for 12 s, and immediately cooled down with cold running water. The wall specimens were clamped under a constant force of 8.2 g and incubated in 20 mM sodium acetate buffer (pH 4.5), which was replaced by the same buffer supplemented with 50 μg ml^–1^ GH5 family endoglucanase at the time of 20 min.

### Hypocotyl dry mass per unit length measurement

Three-day-old Arabidopsis etiolated seedlings were collected. The hypocotyl segments were made in a group of 15 seedlings aligned on a glass slide. The lengths of hypocotyl segments were recorded for individual groups of seedlings. The hypocotyl segments were submerged in ddH_2_O, frozen at –80 °C, freeze-dried, and weighed. The dry mass per unit length was calculated as dry mass/length (μg mm^–1^).

### Oryzalin treatment

Wild-type seeds were grown in the dark for 3 d on a half-strength MS plate without sucrose and supplemented with 300 nM oryzalin. Oryzalin was dissolved in DMSO to create stock solutions.

### Apoplastic pH measurement

Seeds were grown on half-strength MS plates without sucrose for 4 d in the dark. A group of 30 intact Arabidopsis seedlings were incubated in depletion medium with 1 μM IAA or 0.02% ethanol solvent control. The pH of the incubation medium was measured using a pH meter before and after 150 min of incubation (Cleland, 1973).

The shoot apexes and roots of 3-day-old Arabidopsis etiolated seedlings were removed using a razor blade. The hypocotyl segments were incubated in depletion medium (10 mM KCl, 1 mM MES, pH 6.0) supplemented with 1 μM IAA or 2 μM FC. Images of both red fluorescent protein (RFP) and green fluorescent protein (GFP) channels were taken at 0 min and 150 min of IAA or FC treatment. Image analysis was performed using ImageJ software. The cell wall area was selected based on the RFP channel and this area was used as an ROI. The mean intensity was measured in the ROI for both GFP and RFP channels (Fendrych *et al.*, 2016).

## Results

### The *csi1-3* mutants display cell expansion defects

**Fig. 1.**
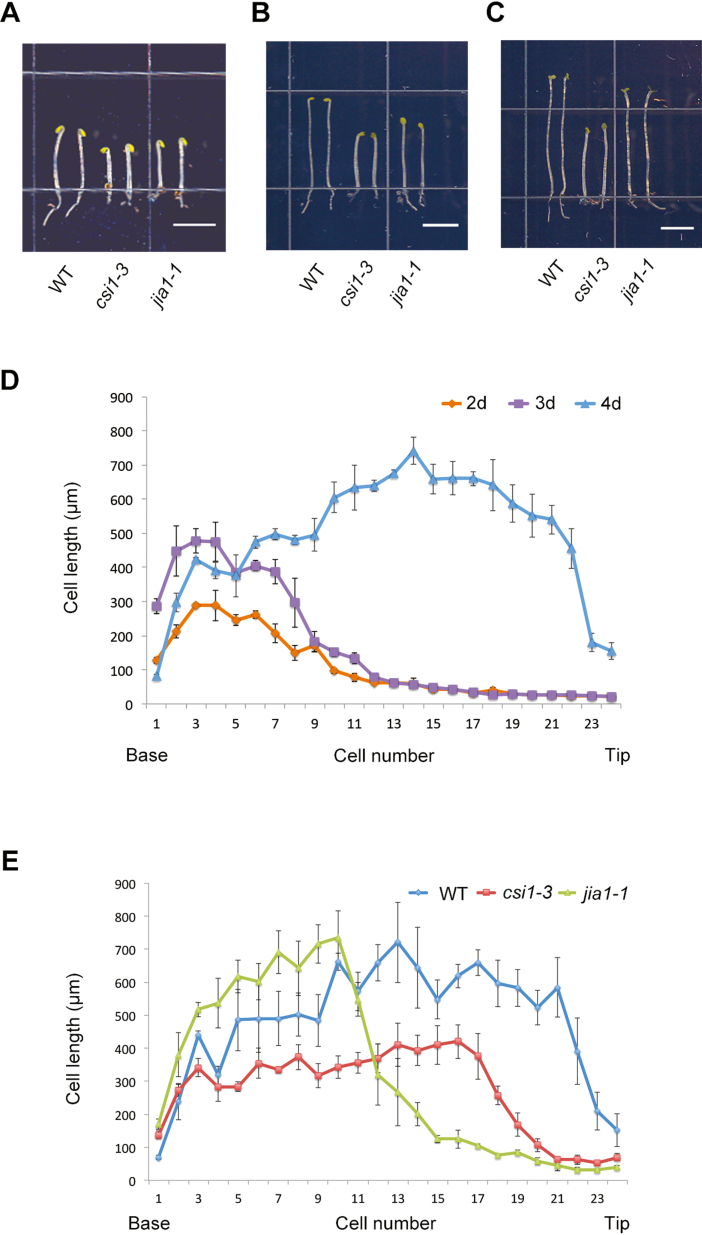
Morphology of *csi1-3* and *jia1-1* mutants. (A) Three-day-old dark-grown seedlings of the WT (Col-0) and *csi1-3* and *jia1-1* mutants (scale bar=5 mm). (B) Four-day-old dark-grown seedlings of the WT (Col-0) and *csi1-3* and *jia1-1* mutants (scale bar=5 mm). (C) Ten-day-old dark-grown seedlings of the WT (Col-0) and *csi1-3* and *jia1-1* mutants (scale bar=5 mm). (D) Measurement of cell length of epidermal cells in WT (Col-0) etiolated seedlings grown for 2, 3, and 4 d. Error bars represent the SE. (E) Measurement of cell length of epidermal cells in a file from the base to the top of 4-day-old, dark-grown hypocotyls of the WT (Col-0), *csi1-3*, and *jia1-1*. Error bars represent the SEM.

To understand the relationship between cellulose microfibril organization and cell elongation, we used *jia1-1* and *csi1-3* mutants that disrupt cellulose biosynthesis. Dark-grown Arabidopsis hypocotyls are a widely used model system to study cell growth (Gendreau *et al.*, 1997; Derbyshire *et al.*, 2007). *csi1-3* was previously shown to have reduced elongation by 40% in dark-grown hypocotyls, and the reduction of the growth rate was maximal at 3–5 d after cold stratification (Gu *et al.*, 2010). We compared growth morphology of 3-day-old dark-grown hypocotyls in the two mutants. Both *csi1-3* and *jia1-1* exhibited dwarf hypocotyls (Fig. 1A). Similar to WT hypocotyls, both *csi1-3* and *jia1-1* had ~22 (±2) epidermal cells from the base to the apical hook (Fig. 1E). As growth in dark-grown hypocotyls does not involve cell division, the reduced hypocotyl length reflects a reduced length of epidermal cells. In dark-grown hypocotyls, epidermal cells elongate in an acropetal fashion in which growth is initiated in the base of the hypocotyl and the elongation zone moves up with time (Gendreau *et al.*, 1997). Compared with the WT, *jia1-1* mutants had reduced cell length in cells #10–24 (Fig. 1E). By day 10 after germination, dark-grown hypocotyls reach their maximum lengths (Gendreau *et al.*, 1997; Gu *et al.*, 2010). WT and *jia1-1* hypocotyls had approximately similar lengths by 10 d post-germination, indicating that *jia1-1* hypocotyls had a delay in acropetal growth acceleration and eventually caught up with WT hypocotyls. Three- and 4-day-old *csi1-3* hypocotyls elongated less than WT hypocotyls and they did not catch up by 10 d post-germination (Fig. 1A–C). These data suggest that reduced cell elongation in *csi1-3* appeared to be independent of its position or time.

### The *csi1-3* mutants are deficient in auxin- or FC-induced hypocotyl elongation

**Fig. 2.**
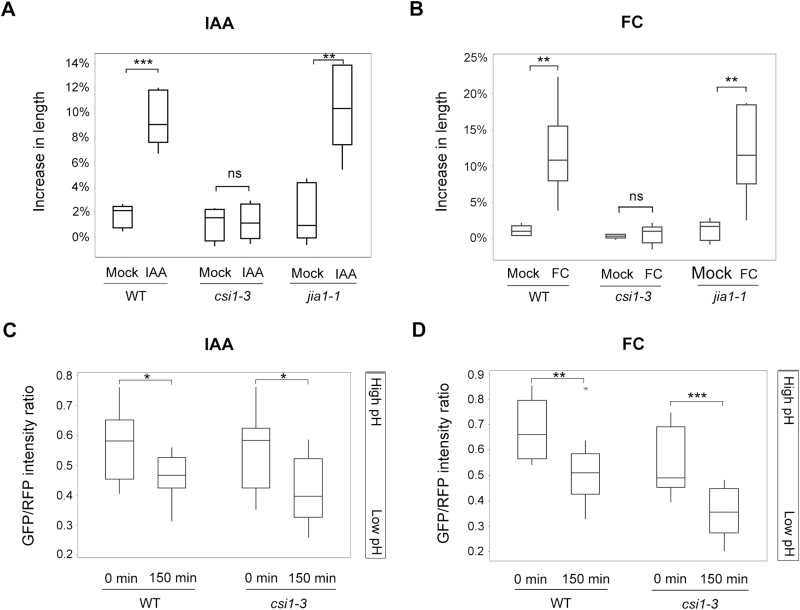
The *csi1-3* mutants are deficient in auxin- or FC-induced hypocotyl elongation. (A) Auxin-induced hypocotyl segment elongation of the WT (Col-0) and *csi1-3* and *jia1-1* mutants. One out of three reproducible independent experiments was used in the analysis. The lengths of the hypocotyls were measured at 0 min and 150 min of each treatment. **P<0.01, ***P<0.001 (*n*=5 in mock treatment for each genotype, *n*=6 in IAA treatment for each genotype). Statistical analysis was performed by two-tailed Student’s *t*-test. (B) FC-induced hypocotyl segment elongation of the WT (Col-0) and *csi1-3* and *jia1-1* mutants. One out of three reproducible independent experiments was used in the analysis. The lengths of the hypocotyls were measured at 0 min and 150 min of each treatment. **P<0.01 (*n*=5 in mock treatment for each genotype, *n*=6 in FC treatment for each genotype). Statistical analysis was performed by two-tailed Student’s *t*-test. (C) Apoplastic pH change in hypocotyl segments of the WT (Col-0) and *csi1-3* in response to IAA treatment. Images were taken at 0 min and 150 min of IAA treatment. The apoplastic pH was reflected by the GFP/RFP intensity ratio. *P<0.05 (*n*=12 individual hypocotyl segments for each genotype). Statistical analysis was performed by two-tailed Student’s *t*-test. (D) Apoplastic pH change in hypocotyl segments of the WT (Col-0) and *csi1-3* in response to FC treatment. Images were taken at 0 min and 150 min of FC treatment. The apoplastic pH was reflected by the GFP/RFP intensity ratio. **P<0.01, ***P<0.001 (*n*=12 individual hypocotyl segments for each genotype). Statistical analysis was performed by two-tailed Student’s *t*-test.

In the classic acid growth hypothesis, cell elongation is induced by acidification of thick extension-limiting outer epidermal cell walls, which can be achieved by either the growth hormone auxin (IAA) or the phytotoxin FC (Rayle and Cleland, 1970, 1977, 1992; Hager *et al.*, 1971; Rayle, 1973; Marre, 1979). Both IAA and FC induce decreases in apoplastic pH, which in turn activates wall-loosening agents such as expansin, resulting in cell wall modification/expansion (McQueen-Mason *et al.*, 1992; Kutschera, 1994; Cosgrove, 1997, 2000). As an approach to understand the control of axial cell elongation in living cells, we adopted a hypocotyl segment system to test how *csi1-3* and *jia1-1* respond to exogenous IAA (Gray *et al.*, 1998; Cosgrove *et al.*, 2002; Takahashi *et al.*, 2012; Fendrych *et al.*, 2016; Spartz *et al.*, 2017). To deplete endogenous auxin, shoot apexes and roots were removed from 3-day-old etiolated Arabidopsis seedlings. To find a minimum IAA concentration that induces sufficient hypocotyl segment elongation, we tested a series of exogenous IAA concentrations and chose 1 μM IAA for the following experiments (Supplementary Fig. S2A). It has been reported that auxin-induced hypocotyl segment elongation occurred after ~20 min of auxin application and lasted ~2 h (Schenck *et al.*, 2010; Takahashi *et al.*, 2012; Fendrych *et al.*, 2016). Thus, we recorded the final length of the hypocotyl segment at 150 min after the application of IAA. The hypocotyl segment corresponds to cells #5–17. The auxin-induced hypocotyl segment elongation was ~50% of the elongation rate of intact hypocotyls under normal growth conditions (Supplementary Fig. S3). The results showed that exogenous auxin induced similar elongation of the hypocotyl segment of the WT and the *jia1-1* mutants, while hypocotyl elongation was impaired in the *csi1-3* mutants (Fig. 2A; Supplementary Fig. S3B). The elongation rate for the auxin-induced hypocotyl segment was similar for the WT (96.6±7.5 µm h^–1^) and *jia1-1* (96.3±17.1 µm h^–1^), but was significantly reduced in *csi1-3* (22.5±9.8 µm h^–1^).

To further verify that *csi1* was not responsive to acid-induced growth, we used the fungal toxin FC, which activates the plasma membrane (PM) H^+^-ATPase independently of the auxin-mediated mechanism (Rayle and Cleland, 1992; Baunsgaard *et al.*, 1998). We found that 2 μM FC was sufficient to trigger the growth of WT hypocotyls at comparable levels to that of 1 μM IAA (Supplementary Fig. S2B). Thus, this concentration was used in the following assays. The hypocotyl segment growth triggered by FC has been observed to occur continuously within 2 h (Schenck *et al.*, 2010; Takahashi *et al.*, 2012; Fendrych *et al.*, 2016). Therefore, we chose to measure the final length of hypocotyls 150 min after FC treatment. *csi1-3* mutants were not responsive to FC-induced hypocotyl elongation, whereas the WT and *jia1-1* mutants exhibited normal responses (Fig. 2B).

### Cell wall acidification is normal in the *csi1-3* mutants

To examine whether cell wall acidification occurs normally in the *csi1-3* mutants, we used a classic method to measure the apoplastic pH of Arabidopsis etiolated seedlings. The pH of the incubation medium was measured, from which cell wall acidification can be inferred (Cleland, 1973). We used approximately thirty 4-day-old intact etiolated seedlings in each measurement. After 150 min of incubation with 1 μM IAA, the pH of the incubation medium dropped from 6.05 (±0.02) to 5.88 (±0.08) in the WT and from 6.05 (±0.02) to 5.87 (±0.07) in *csi1-3* mutants (Supplementary Table S1). In the presence of IAA, the pH dropped similarly for both the WT and *csi1-3*. However, the change of pH is not statistically significant for both the WT and *csi1-3* upon IAA incubation. It is possible that intact Arabidopsis hypocotyls did not excrete hydrogen ions efficiently as compared with segments of *Avena* coleoptiles (Cleland, 1973).

The apoplastic pH sensor apo-pHusion was recently developed to allow direct measurement of apoplastic pH changes in epidermal cells (Gjetting *et al.*, 2012; Fendrych *et al.*, 2016). We crossed *csi1-3* with the line containing the apo-pHusion sensor, which was a tandem fusion of two fluorescent proteins, monomeric RFP1 (mRFP1) and enhanced GFP (EGFP). A chitinase-targeting sequence was added in the construct to target the sensor to the apoplast (Gjetting *et al.*, 2012). mRFP1 is considered to be insensitive to pH changes in the physiologically relevant range, whereas EGFP is sensitive to pH changes and low pH inhibits the EGFP fluorescence (Gjetting *et al.*, 2012). The hypocotyl segments of the WT and *csi1-3* mutants were incubated in depletion medium supplemented with IAA or FC. Images of both RFP and GFP channels were taken at 0 min and 150 min of IAA and FC treatment. The ratio of GFP/RFP fluorescent intensity was measured and calculated in Image J, where RFP intensity was used as an intramolecular reference. The results showed that in both the WT and *csi1-3* mutants, the GFP/RFP intensity ratio dropped after IAA or FC treatment, indicating a drop in apoplastic pH and normal apoplastic acidification (Fig. 2C, D). The normal acidification in *csi1-3* indicates that the loss of auxin-induced cell elongation in *csi1-3* is not due to deficient apoplastic acidification.

### The *csi1-3* mutants have less extensible cell walls

**Fig. 3.**
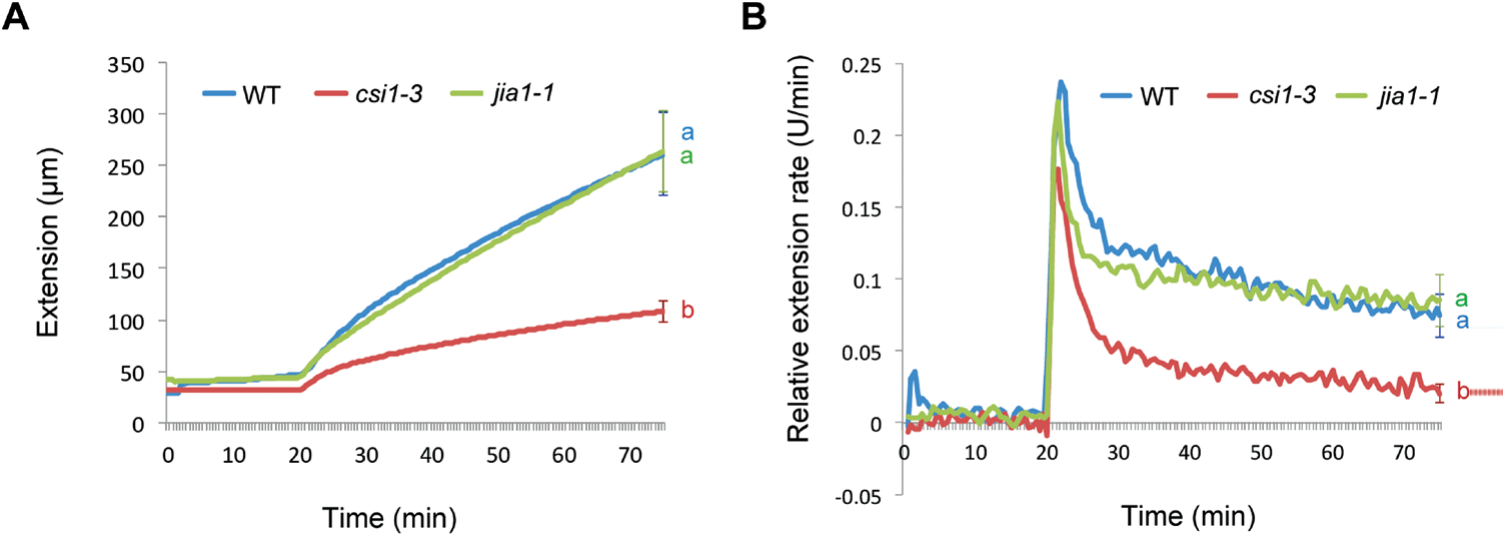
The *csi1-3* mutants exhibit less wall extensibility. (A) Extension of native walls in response to acidic buffer (pH 4.5). The samples were 3-day-old dark-grown seedlings of the WT (Col-0) and *csi1-3* and *jia1-1* mutants. One out of three reproducible independent experiments was used in the analysis. Each curve is an average of eight individual responses. Error bars indicate the SEM. Lower case letters denote statistically significant differences, *P*<0.01 (*n*=8 for each genotype). Statistical analysis was performed using one-way ANOVA with Tukey test. (B) Relative extension rate of native walls in response to acidic buffer (pH 4.5). The samples were 3-day-old dark-grown seedlings of the WT (Col-0) and *csi1-3* and *jia1-1* mutants. One out of three reproducible independent experiments was used in the analysis. Each curve is an average of eight individual responses. Error bars indicate the SEM. Lower case letters denote statistically significant differences, *P*<0.01 (*n*=8 for each genotype). Statistical analysis was performed using one-way ANOVA with Tukey test.

The cell wall creep assay has been used to mimic the slow creep of cell walls during cell expansion (Cosgrove, 1998; Durachko and Cosgrove, 2009; Park and Cosgrove, 2012*b*). We measured the acid-induced creep responses of native walls. To kill the cells, 3-day-old etiolated seedlings were frozen and thawed prior to the experiment. An entire seedling was clamped at a constant force (2.5 g) in pH 6.8 buffer. After 20 min incubation, the buffer was replaced with pH 4.5 buffer. For each genotype, creep responses of eight individual seedlings were recorded every 30 s for 75 min. The walls of the WT and *jia1-1* mutants extended rapidly in response to acidic buffer. In contrast, the wall extension and relative extension rate of the *csi1-3* mutants was ~41% and 27% of that of WT hypocotyls, respectively (Fig. 3A, B). Although both *csi1-3* and *jia1-1* had reduced cell elongation in 3-day-old de-etiolated hypocotyls *in vivo*, only *csi1-3* had a diminished creep response to acidic buffers. These results support the idea that different mechanisms account for cell expansion defects in the two mutants.

The larger diameter of the *csi1-3* hypocotyls might result in reduced stress (=force divided by the wall cross-sectional area). Because it is technically difficult to measure the wall cross-sectional area of the hypocotyl (little of the hypocotyl cross-section is occupied by cell wall), we used dry mass per unit length of hypocotyl segments (µg mm^–1^) as a proxy (Cleland, 1967) (Supplementary Table S2). Our results showed that *jia1-1* (1.47±0.18) had a similar dry mass per unit length to the WT (1.75±0.42). In contrast, *csi1-3* had a higher dry mass per unit length (5.26±2.11), but this was not a statistically significant difference. Nevertheless, the results do suggest that less stress may have been applied to *csi1-3* as a result of the greater diameter of the hypocotyls.

We next tested whether *csi1* affects an endoglucanase-induced creep response as a means to test the wall’s ability to undergo creep. It was shown previously that a creep response was induced by PpXG5, a family-5 endoglucanase (Park and Cosgrove, 2012*b*). The GH5 family endoglucanase displays hydrolytic activity towards both xyloglucan and disordered cellulose (Park and Cosgrove, 2012*b*). To inactivate endogenous enzymes and expansins, samples were boiled for 12 s and immediately cooled down with cold running water. Because Arabidopsis hypocotyls were not amenable to heat inactivation followed by creep experiments, we used leaf petiole instead. The leaf petioles were boiled to inactivate endogenous wall-loosening enzymes and abraded to promote enzyme penetration through the cuticle. The wall specimens were clamped at a constant force (8.2 g) and 50 μg ml^–1^ GH5 family endoglucanase was added after 20 min of incubation. The *csi1-3* samples showed a small creep rate compared with that in the WT (Supplementary Fig. S4). Diminished creep responses in *csi1* suggest that the reduced wall extensibility in *csi1-3* is likely to be due to changes in wall architecture.

### The *csi1-3* mutants display altered cellulose microfibril organization

**Fig. 4.**
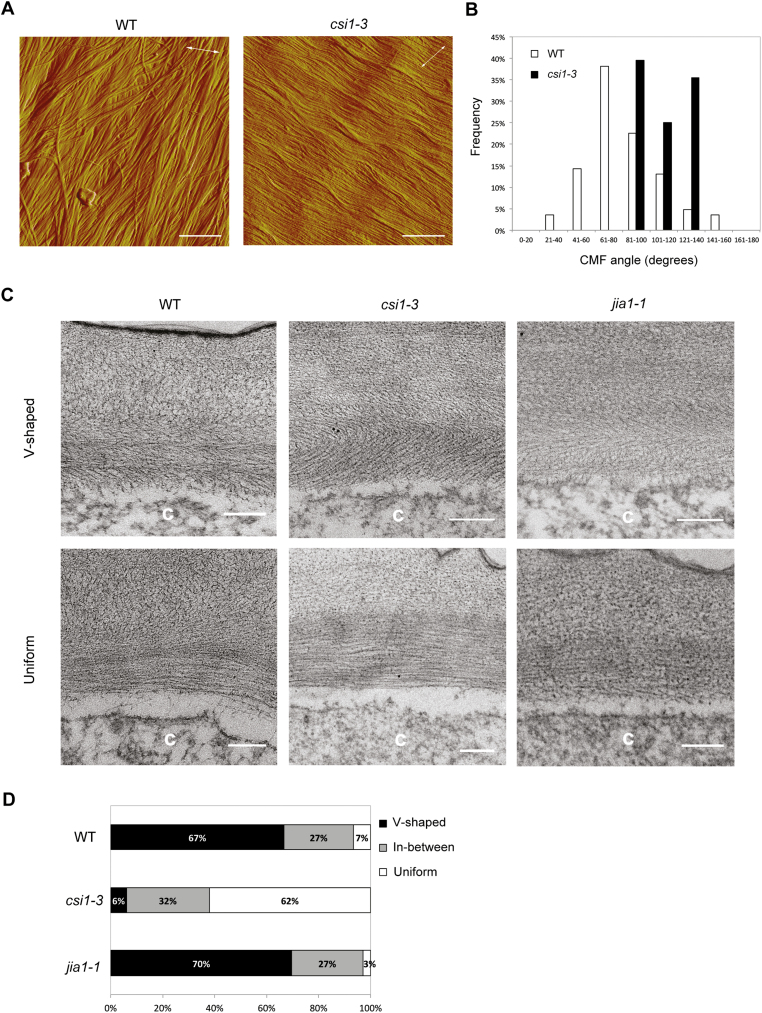
Cellulose microfibril (CMF) organization is altered in the *csi1-3* mutants. (A) AFM images of 3-day-old dark-grown seedlings of the WT (Col-0) and *csi1-3* mutant. The long axes of the cells are indicated by white double-headed arrows (scale bars=400 nm). (B) Quantitative analysis of CMF angles in AFM images. *n*=84 CMFs in 28 AFM images from 13 cells for the WT, *n*=48 CMFs in 16 AFM images from seven cells for *csi1-3*. (C) TEM images of 3-day-old dark-grown seedlings of the WT (Col-0) and *csi1-3* and *jia1-1* mutants (scale bars=200 nm). The letter ‘c’ on the TEM images indicates the cytosol of the cell. (D) Quantitative analysis of the TEM images. *n*=30 TEM images from three individual seedlings for each genotype. V-shaped refers to the ‘herringbone’ pattern of the cell wall. Uniform refers to unifying parallel orientation of polysaccharides. In between is a category that has mixed features of a V-shaped and uniform cell wall.

It is commonly accepted that the direction of maximal expansion depends on the direction of the alignment of cellulose microfibrils. To assess whether the reduced cell elongation in *csi1-3* and *jia1-1* is correlated with misalignment of cellulose microfibrils, we examined the organization of cellulose microfibrils. AFM was used to examine the organization of the most recently deposited cellulose microfibrils in outer periclinal walls in epidermal cells of Arabidopsis hypocotyls (Supplementary Fig. S1). The hypocotyl samples were kept in water during sample preparation and imaging, so the organization of cellulose microfibrils was maintained in a near-native state (Zhang *et al.*, 2016). AFM images revealed that the majority of most recently deposited cellulose microfibrils in the outer periclinal walls were arranged in transverse orientation perpendicular to the growth axis in WT seedlings (Fig. 4A, B). Although the majority of cellulose microfibrils were deposited in transverse orientation in *csi1-3* mutants, there was less variation in orientation of cellulose microfibrils as compared with the WT (Fig. 4A, B). A similar parallel alignment of cellulose microfibrils was previously shown for *jia1-1* (Lei *et al.*, 2014). *csi1-3* and *jia1-1* mutants exhibited different cell expansion abilities but similar parallel alignment of the most recently deposited cellulose microfibrils, indicating that cellulose microfibril alignment in the innermost outer periclinal wall alone is insufficient to regulate axial cell elongation.

### The crossed-polylamellate architecture of the outer epidermal cell wall is lost in the *csi1-3* mutants

It has been hypothesized that the outer epidermal wall represents the growth-limiting structure of the multicellular system in hypocotyls (Kutschera, 1992). We therefore examined the structure of the outer epidermal wall using TEM. Polysaccharides in oblique sections of 3-day-old Arabidopsis etiolated hypocotyl segments were stained with periodic acid thiocarbohydrazide–silver proteinate (PATA) and lead citrate. In WT cells, epidermal cell walls have a crossed-polylamellate architecture in which cellulose microfibrils are relatively well aligned within each lamella, but the orientation varies between successive lamellae (Fig. 4C, D). Occasionally outer epidermal walls show uniform parallel orientation of polysaccharides across multiple lamellae in the WT (7%). A comparable texture was observed for *jia1-1* (Fig. 4C, D). In contrast, the majority of outer epidermal walls (62%) showed uniform parallel orientation of polysaccharides in different lamellae in *csi1-3* (Fig. 4C, D).

### Pharmacological disruption of microtubules phenocopies loss of auxin- and fusicoccin-induced cell expansion and loss of the crossed-polylamellate wall architecture in *csi1-3*

CSI1 was previously shown to be required for the co-alignment between CSC trajectories and cortical MTs (Gu *et al.*, 2010; Li *et al.*, 2012). In *csi1-3*, CSC trajectories did not follow the underlying microtubules in epidermal cells ~0.5 mm below the apical hook (Li *et al.*, 2012). As the cell expansion defect is not limited to the upper part of the hypocotyl, we carefully examined dynamics of yellow fluorescent protein (YFP)–CESA6 and cortical MTs in all regions of hypocotyls. In WT seedlings, MT orientation and CSC trajectories were consistently aligned across different regions, from predominantly transversely oriented in cells #17–21, to oblique orientation in cells #9–13, to longitudinal orientation in cells #1–5. In contrast, *csi1-3* seedlings displayed consistently transverse orientation of CSC trajectories in all regions, while MT orientation was similar to that of WT seedlings (Supplementary Fig. S5). The angles of MT or CSC tracks against the horizontal direction were measured using FibrilTool, an ImageJ plug-in to quantify fibrillar structures in microscopy images (Boudaoud *et al.*, 2014), further supporting the conclusion that the average trajectories of YFP–CESA6 and MTs were uncoupled in *csi1-3* (Li *et al.*, 2012; Supplementary Fig. S5).

**Fig. 5.**
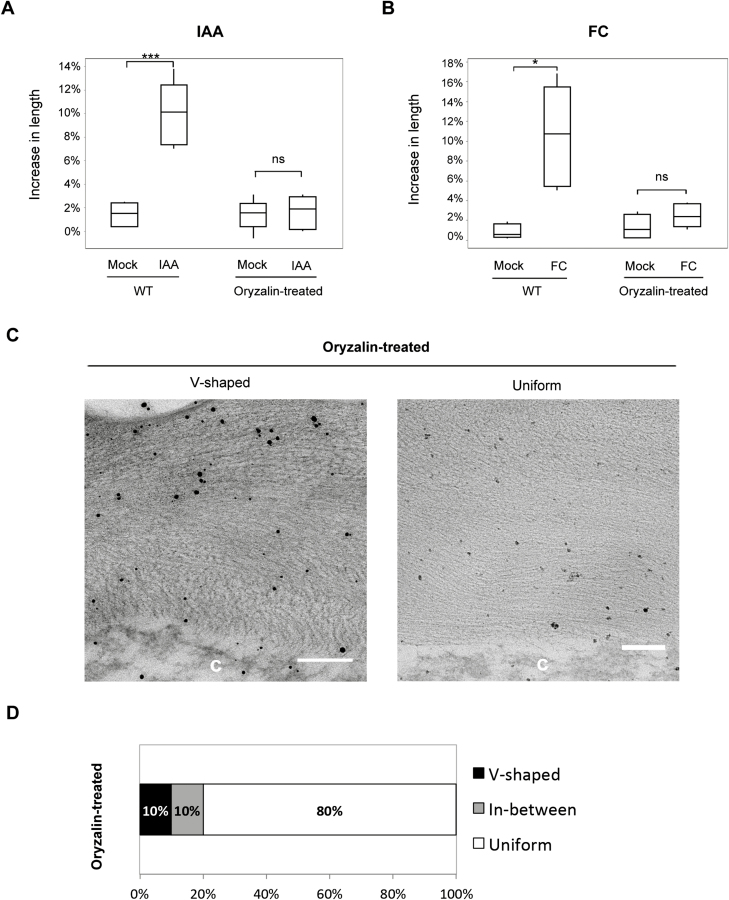
Pharmacological disruption of microtubules results in loss of the crossed-polylamellate wall architecture. (A) Auxin-induced hypocotyl segment elongation of the WT (Col-0) treated with 300 nM oryzalin. One out of three reproducible independent experiments was used in the analysis. The lengths of the hypocotyls were measured at 0 min and 150 min of each treatment. ***P<0.001 (*n*=6 in each treatment for both WT and oryzalin-treated seedlings). Statistical analysis was performed by two-tailed Student’s *t*-test. (B) FC-induced hypocotyl segment elongation of the WT (Col-0) treated with 300 nM oryzalin. One out of three reproducible independent experiments was used in the analysis. The lengths of the hypocotyls were measured at 0 min and 150 min of each treatment. *P<0.05 (*n*=5 in each treatment for both the WT and oryzalin-treated seedlings). Statistical analysis was performed by two-tailed Student’s *t*-test. (C) TEM images of 3-day-old dark-grown seedlings of the WT (Col-0) treated with 300 nM oryzalin (scale bars=200 nm). The letter ‘c’ on the TEM images indicates the cytosol of the cell. (D) Quantitative analysis of the TEM images. *n*=30 TEM images from three individual seedlings for each treatment. V-shaped refers to the ‘herringbone’ pattern of the cell wall. Uniform refers to unifying parallel orientation of polysaccharides. In between is a category that has mixed features of a V-shaped and uniform cell wall.

The loss of crossed-polylamellate walls in *csi1-3* is consistent with the hypothesis that crossed-polylamellate walls of the outer epidermal cell require MT-dependent rotation of cellulose microfibril deposition (Chan *et al.*, 2007, 2010). To further explore the role of MTs in cell expansion, we treated WT seedlings with oryzalin, an MT depolymerization drug. We chose an oryzalin concentration that mimicked the morphology of *csi1-3* in terms of the cell expansion defects (Li *et al.*, 2012). Oryzalin-treated hypocotyls did not elongate in response to auxin or FC treatments, thus mimicking the response of *csi1-3* (Fig. 5A, B). Moreover, TEM images of oryzalin-treated seedlings also showed a loss of the crossed-polylamellate wall architecture (Fig. 5C, D). The majority of oryzalin-treated seedlings displayed a uniform parallel orientation of polysaccharides (24/30 images). These results suggest that MTs play an important role in the formation of the crossed-polylamellate wall architecture, and loss of auxin-induced cell elongation accompanies loss of crossed-polylamellate construction.

## Discussion

### Crossed-polylamellate cell wall architecture

Crossed-polylamellate walls are often observed in rapidly growing epidermal cells in both hypocotyls and roots (Chafe and Wardrop, 1972; Roland *et al.*, 1977; Takeda and Shibaoka, 1981; Hodick and Kutschera, 1992). It is postulated that different mechanisms account for the crossed-polylamellate wall feature in two systems (Li *et al.*, 2014). In Arabidopsis hypocotyls, cyclic reorientation of MTs is accompanied by a corresponding reorientation of CSC trajectories. The dynamics of the cortical MT pattern actively dictate the orientation of cellulose microfibril deposition. The rotation of CSC trajectories was blocked by either stabilizing or depolymerizing MTs, and this resulted in loss of crossed-polylamellate cell wall patterns (Chan *et al.*, 2010). The cyclic reorientation appears to be shoot specific as microtubules do not rotate in roots in Arabidopsis. In Arabidopsis roots, there is a one-way microfibril reorientation from transverse to longitudinal as epidermal cells elongate (Liang *et al*., 1996; Sugimoto *et al.*, 2000; Granger and Cyr, 2001; Anderson *et al.*, 2010). The one-way microfibril reorientation is most probably a passive process independent of MTs. Consistent with the idea that the shoot and root adopt different mechanisms in growth controls, auxin promotes cell expansion in aerial tissues but inhibits cell expansion in roots at concentrations >10^–8^ M (Taiz, 1984; Ishikawa and Evans, 1993). *csi1* resulted in the loss of CSC trajectory re-orientation and loss of crossed-polylamellate cell wall patterns, supporting the idea of an MT-dependent mechanism in hypocotyls (Lloyd, 2011). Further supporting the MT-dependent mechanism, the removal of MTs by oryzalin phenocopies the loss of crossed-polylamellate cell wall patterns in *csi1*.

### Correlation between loss of crossed-polylamellate wall architecture and acid-induced growth

Auxin-induced growth is characterized by activation of PM proton pumps that leads to acidification of the cell wall. The acidification of apoplastic space activates expansin that loosens the connection between cellulose microfibrils and non-cellulosic polysaccharides. As a result, cell expansion occurs. *csi1* has defects in auxin- or FC-induced growth while the apoplastic acidification is normal, suggesting that the defect may reside in wall loosening. Indeed, *csi1* walls are less extensible in creep assays. As wall creep mimics wall enlargement during cell growth, these results confirm a correlation between defects in wall mechanics and loss of acid-induced growth in *csi1*. Because pharmacological disruption of MTs phenocopies loss of auxin- and FC-induced cell expansion and loss of the crossed-polylamellate wall architecture in *csi1*, we reasoned that MTs play an important role in the formation of the crossed-polylamellate wall and are dependent on CSI1. In addition, the correlation between loss of crossed-polylamellate wall and loss of auxin- and FC-induced cell growth relies on both MT and CSI1 function.

Previous results showed that xyloglucan-deficient *xxt1 xxt2* mutants had diminished responses to FC-induced growth and diminished creep responses to acid buffers (Park and Cosgrove, 2012*a*). Interestingly, the wall architecture is similar to that of *csi1*, both displaying well aligned transversely oriented cellulose microfibrils in the innermost wall and partial loss of the crossed-polylamellate wall (Xiao *et al.*, 2016). Xyloglucan is speculated to reduce interactions between cellulose microfibrils and reduce their bundling (Xiao *et al.*, 2016). It is unclear whether the defects in wall mechanics and/or loss of acid-induced growth in *xxt1 xxt2* alter MT organization or CSI1 function. TEM analysis revealed an overall appearance of loss of the crossed-polylamellate wall in *xxt1 xxt2* and *csi1* but it cannot discern molecular difference such as bundle sizes, microfibril length, connection between different lamellae, microfibril motions, and the incorporation of other cell wall polymers. Precise measurements are required to determine how such cell wall parameters affect the overall wall organization and wall mechanical properties in *xxt1 xxt2* and *csi1*. The mechanism by which cell wall architecture affects acid growth is unclear. Potential mechanisms may involve the inaccessibility of expansin to specific wall-loosening sites, changes in interconnectivity between cellulose microfibrils and other cell wall polymers, and defects in the mechanism of cellulose microfibril sliding or slippage (Cosgrove, 2018).

### Is organization of cellulose microfibrils dependent on microtubules?

The ‘direct guidance model’ postulates that MTs guide the deposition of cellulose microfibrils (Heath, 1974). The observation that the CSCs trajectories were uncoupled from MTs in *csi1* supports the direct guidance model. With the discovery of the linker protein CSI1, a modified guidance model is proposed in which MTs guide the deposition of cellulose microfibrils through direct attachment mediated by CSI1 (Gu *et al.*, 2010; Li *et al.*, 2012). As CSC trajectories are proxies for the orientation of nascent cellulose microfibrils, it is predicted that cellulose microfibrils would have disorganized orientations in *csi1*. Surprisingly, the newly deposited cellulose microfibrils in *csi1* mutants are aligned more parallel than those of the WT. These results suggest that the direct guidance model is inadequate to explain the origin of the initial orientation of cellulose microfibrils. A re-visit of alternative models is needed. The self-assembly mechanism suggests that wall formation is a spontaneous process, similar to liquid crystal formation (Neville *et al.*, 1976; Neville, 1985; Vian and Roland, 1987; Vian *et al.*, 1993). Another model takes the density of CSC and geometry of the cell into consideration (Emons and Mulder, 2000; Dyson and Jensen, 2010). The helicoidal wall texture in the root hair was simulated based on the geometrical theory. As Arabidopsis hypocotyls have a crossed-polylamellate wall rather than a helicoidal wall, it remains to be tested whether the geometrical theory holds true in hypocotyls.

### Is microfibril alignment sufficient to explain axial cell elongation?

The observations that MTs and microfibrils in the most recently deposited walls had a dominant orientation perpendicular to the growth axis led to the hypothesis that inner periclinal walls control the growth direction whereas the outer periclinal walls determine the rate of organ elongation (Kutschera, 2008; Crowell *et al.*, 2010, 2011). Despite the differences in response to auxin- and FC-induced acid growth in *csi1* and *jia1*, elongation still occurs in both mutants, consistent with the proposed role of inner periclinal walls controlling the growth direction. However, the most recently deposited cellulose microfibrils in *csi1* and *jia1* were aligned more parallel than those in WT plants. Therefore, the growth defect in *csi1* and *jia1* cannot be simply explained by the disorganization of the microfibrils and the loss of growth anisotropy. Moreover, the growth defect in *csi1* and *jia1* appeared to be controlled by different mechanisms as *csi1* had reduced cell elongation independently of the cell position along hypocotyls, whereas *jia1* had developmental delays in acropetal growth acceleration but eventually was able to reach maximal growth similar to the WT. The synthesis of cellulose is thought to occur simultaneously with elongation to allow continuous deposition of cellulose microfibrils in the innermost layer. Previous experiments have shown that cellulose deposition and net wall polysaccharide synthesis are not tightly linked to the rate of the elongation (Refregier *et al*., 2004; Derbyshire *et al.*, 2007). *jia1* had a much more severe defect in cellulose synthesis as compared with *csi1* (Lei *et al.*, 2014). It is likely that the delay in growth acceleration represents a mechanism to cope with severe cellulose deficiency.

In summary, this study has several significant implications. First, CSI1 is required for the formation of crossed-polylamellate walls as loss of CSI1 resulted in loss of crossed-polylamellate walls. Secondly, the crossed-polylamellate wall architecture is required for auxin-induced acid growth. Thirdly, cellulose microfibrils maintain a default transverse orientation without CSI1, indicating that the orientation of nascent cellulose microfibrils is independent of CESA–MT linkage. Last, microfibril alignment is not sufficient to control axial cell elongation. Our results lay a foundation for future studies on the mechanistic role of wall architecture during acid growth.

## Supplementary data

Supplementary data are available at *JXB* online.

Fig. S1. A cartoon of the cross-section of an Arabidopsis hypocotyl.

Fig. S2. Auxin- and FC-induced hypocotyl elongation.

Fig. S3. Measurement of hypocotyl elongation rate.

Fig. S4. The *csi1-3* mutants have impaired endoglucanase-induced creep response.

Fig. S5. CSC trajectories and cortical MTs are uncoupled in the *csi1-3* mutant.

Table S1. Cell wall acidification of the wild type (Col-0) and *csi1-3* mutant.

Table S2. Dry mass per unit length measurement of the wild type (Col-0), and *csi1-3* and *jia1-1* mutants.

eraa063_suppl_Supplementary_Figures_S1-S5_Table_S1-S2
