# Supplementary material for: Cellulose synthase interactive1- and microtubule-dependent cell wall architecture is required for acid growth in Arabidopsis hypocotyls
Source: J Exp Bot. 2020 Feb 4;71(10):2982–94. doi: 10.1093/jxb/eraa063 (PMC7260726; doi:10.1093/jxb/eraa063)
Supplement: eraa063_suppl_Supplementary_Figures_S1-S5_Table_S1-S2 [file eraa063_suppl_supplementary_figures_s1-s5_table_s1-s2.pdf]

CELLULOSE SYNTHASE INTERACTIVE1- and Microtubule-Dependent Cell Wall Architecture Is Required for Acid Growth in *Arabidopsis* Hypocotyls

Xiaoran Xin[1], Lei Lei[1, a], Yunzhen Zheng[2], Tian Zhang[2], Sai Venkatesh Pingali[3], Hugh O'Neill[3], Daniel J. Cosgrove[2], Shundai Li[1*], Ying Gu[1*]

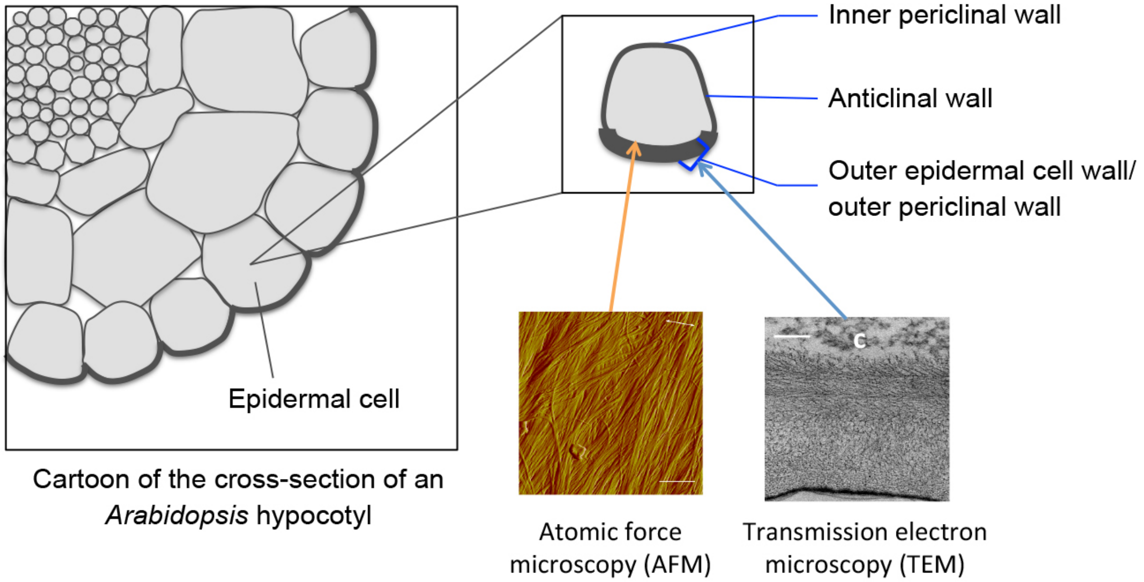

Supplementary Fig. S1. A cartoon of the cross-section of an *Arabidopsis* hypocotyl.
The outer epidermal cell wall is the periclinal wall that faces the external environment of the plant. The corresponding wall visualized by atomic force microscopy (AFM) and transmission electron microscopy (TEM) is shown. AFM examines the organization of the most recently deposited cellulose microfibrils in outer periclinal walls in *Arabidopsis* epidermal cells. TEM displays the structure of the entire outer epidermal wall. The white double-headed arrow on the AFM image indicates the long axis of the cell. The letter "c" on the TEM image indicates cytosol of the cell.

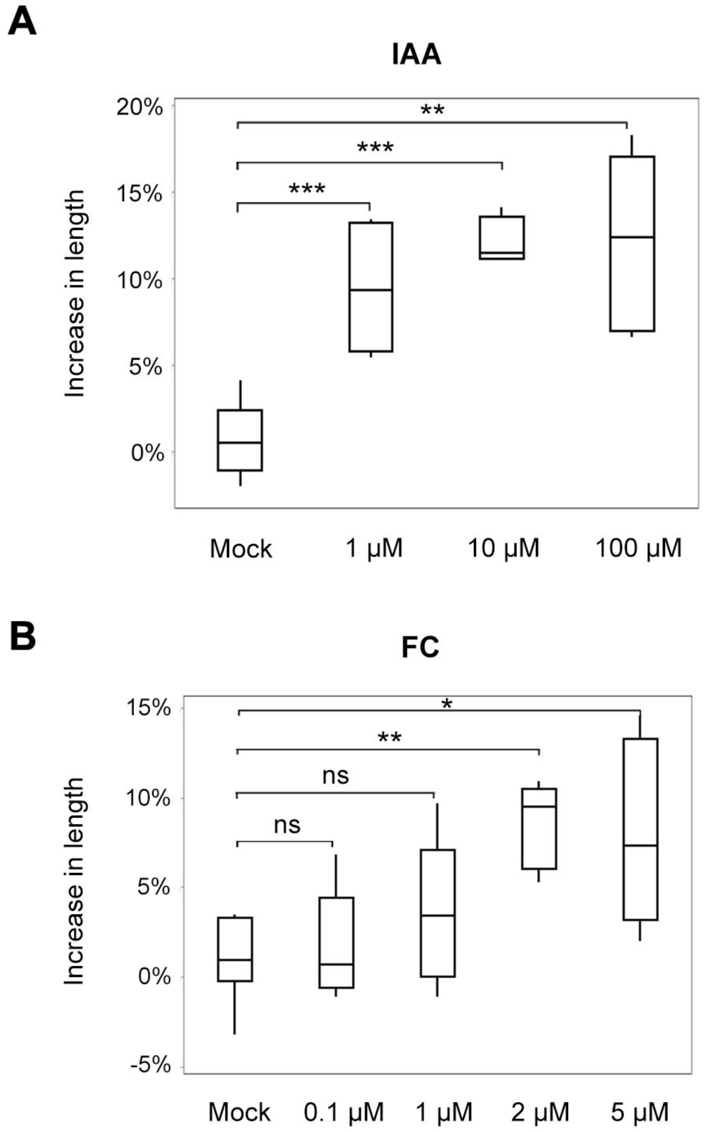

Supplementary Fig. S2. Auxin- and FC- induced hypocotyl elongation.
(A) Dose-response graph of IAA-induced hypocotyl elongation of wild type (Col-0). The lengths of the hypocotyls were measured at 0 min and 150 min of each treatment. Increase in length was calculated as [(length at 150 min – length at 0 min)/ length at 0 min] x 100%. Mock treatment was defined as using 0.02% ethanol (solvent) instead of IAA. ** $P < 0.01$, *** $P < 0.001$ (n = 6 for each treatment). Statistical analysis was performed by two-tailed Student's *t*-test.
(B) Dose-response graph of FC-induced hypocotyl elongation of wild type (Col-0). The lengths of the hypocotyls were measured at 0 min and 150 min of each treatment. Increase in length was calculated as [(length at 150 min – length at 0 min)/ length at 0 min] x 100%. Mock treatment was defined as using 0.02% ethanol (solvent) instead of FC. * $P < 0.05$, ** $P < 0.01$ (n = 6 for each treatment). Statistical analysis was performed by two-tailed Student's *t*-test.

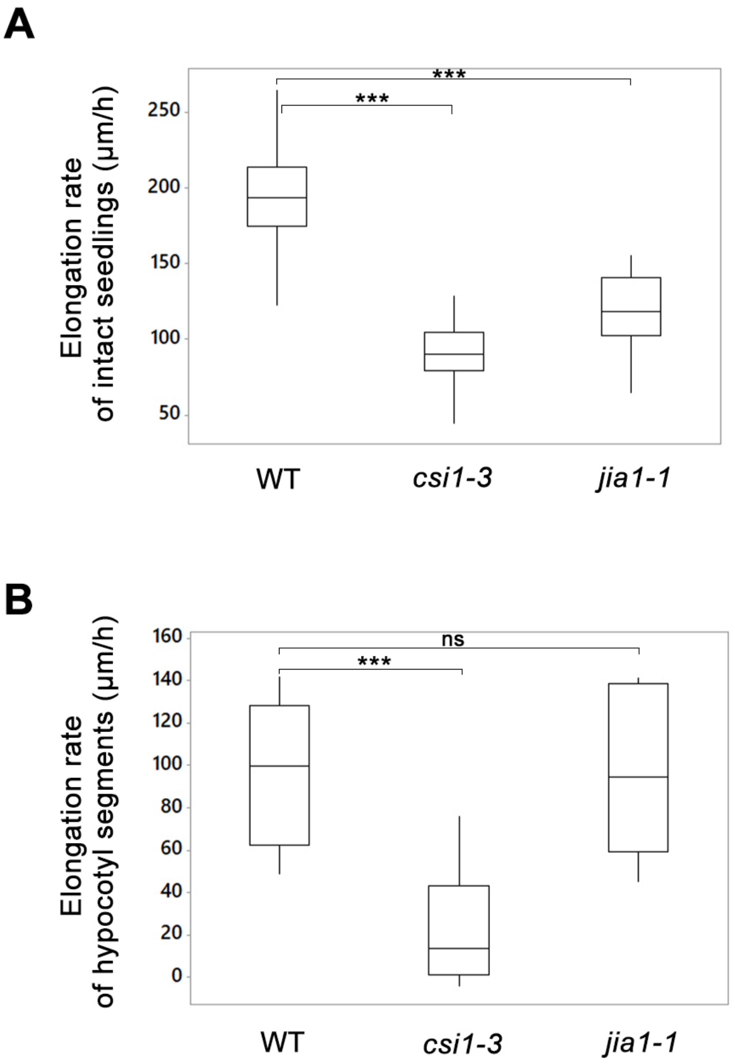

Supplementary Fig. S3. Measurement of hypocotyl elongation rate.
(A) Elongation rate of intact seedlings of wild-type (Col-0), *csi1-3*, and *jia1-1* mutants from day 2 to day 3. Seedlings were grown in the dark on vertical half-strength Murashige and Skoog (MS) plates without sucrose. The lengths of hypocotyls were measured at day 2 and day 3. *** $P < 0.001$ (n = 34 for each genotype). Statistical analysis was performed by two-tailed Student's *t*-test.
(B) Elongation rate of auxin-induced hypocotyl segments of wild-type (Col-0), *csi1-3*, and *jia1-1* mutants. The lengths of the hypocotyls were measured at 0 min and 150 min of 1 µM IAA treatment. *** $P < 0.001$ (n = 12 for each genotype). Statistical analysis was performed by two-tailed Student's *t*-test.

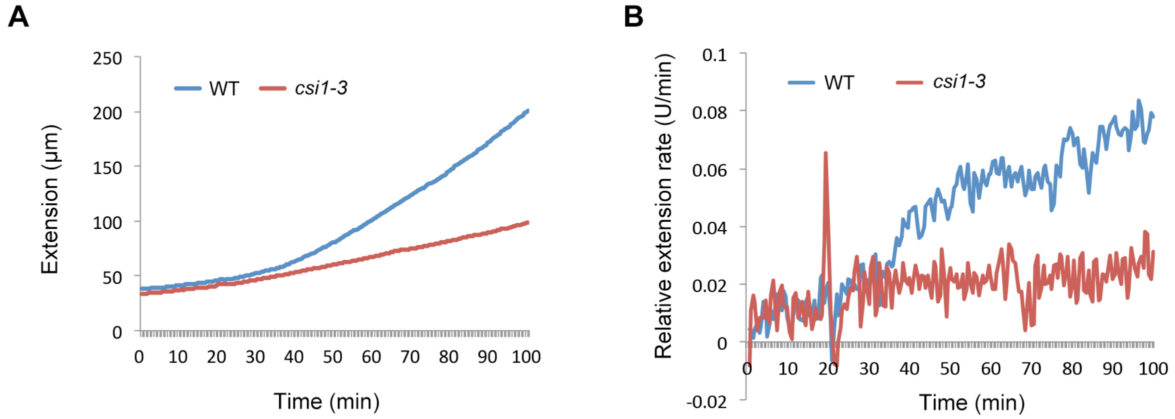

Supplementary Fig. S4. The *csi1-3* mutants have impaired endoglucanase-induced creep response.

(A and B) Extension and relative extension rate of heat-inactivated wild type (Col-0) and *csi1-3* walls in pH 4.5 buffer upon the addition of 50 µg/mL GH5 family endoglucanase at 20 min. The samples were leaf petioles from wild type and *csi1-3* mutant. Each curve is an average of at least four individual responses (n = 4 for wild type, n = 5 for *csi1-3*).

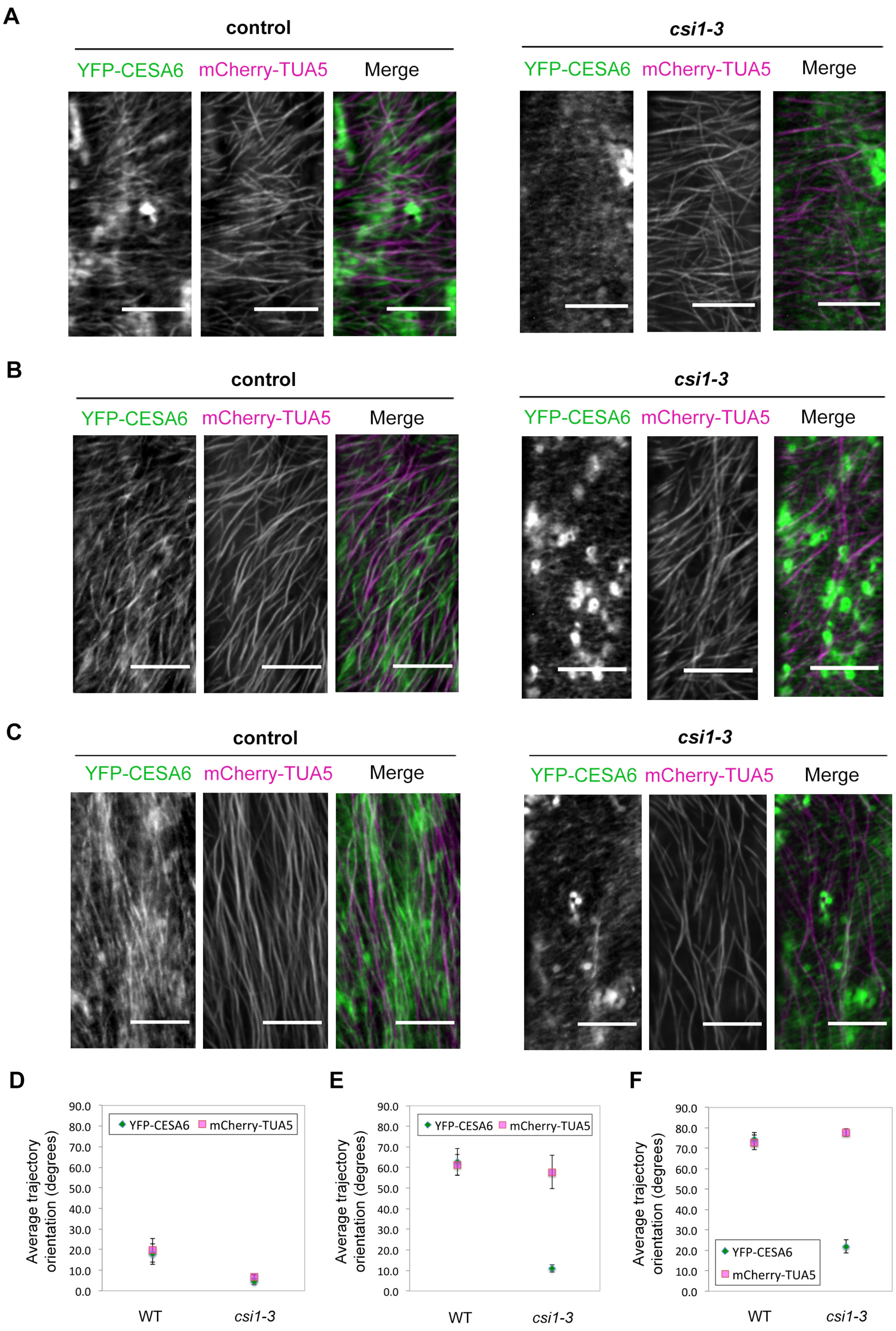
A
control
csi1-3
YFP-CESA6
mCherry-TUA5
Merge
B
C
D
E
F
Average trajectory orientation (degrees)
90.0
80.0
70.0
60.0
50.0
40.0
30.0
20.0
10.0
0.0
WT
csi1-3

Supplementary Fig. S5. CSC trajectories and cortical MTs are uncoupled in *csi1-3* mutant.

(A-C) Two-channel confocal imaging of epidermal cells in 3-d-old etiolated seedlings from control and *csi1-3* mutant expressing YFP-CESA6 and mCherry-TUA5 in cell #17-21 (A), #9-13 (B), and #1-5 (C), respectively. Scale bars, 10 μm.

(D) Analysis of the average trajectory orientation of YFP-CESA6 and mCherry-TUA5 in control and *csi1-3* mutant in cell #17-21. Error bars are SEM. In control, the angles of YFP-CESA6 and mCherry-TUA5 trajectories are 17.87 ± 12.38 (mean ± SD) degrees and 19.67 ± 14.00 (mean ± SD) degrees, respectively. $P > 0.05$ (n = 6 for each group). In the *csi1-3* mutant, the angles of YFP-CESA6 and mCherry-TUA5 trajectories are 4.49 ± 4.00 (mean ± SD) degrees and 6.79 ± 2.49 (mean ± SD) degrees, respectively. $P > 0.05$ (n = 7 for each group).

(E) Analysis of the average trajectory orientation of YFP-CESA6 and mCherry-TUA5 in control and *csi1-3* mutant in cell #9-13. Error bars are SEM. In control, the angles of YFP-CESA6 and mCherry-TUA5 trajectories are 62.84 ± 17.27 (mean ± SD) degrees and 61.22 ± 13.35 (mean ± SD) degrees, respectively. $P > 0.05$ (n = 7 for each group). In the *csi1-3* mutant, the angles of YFP-CESA6 and mCherry-TUA5 trajectories are 11.07 ± 4.82 (mean ± SD) degrees and 57.79 ± 21.40 (mean ± SD) degrees, respectively. $P < 0.01$ (n = 7 for each group).

(F) Analysis of the average trajectory orientation of YFP-CESA6 and mCherry-TUA5 in control and *csi1-3* mutant in cell #1-5. Error bars are SEM. In control, the angles of YFP-CESA6 and mCherry-TUA5 trajectories are 74.29 ± 8.13 (mean ± SD) degrees and 72.95 ± 8.91 (mean ± SD) degrees, respectively. $P > 0.05$ (n = 6 for each group). In the *csi1-3* mutant, the angles of YFP-CESA6 and mCherry-TUA5 trajectories are 21.82 ± 9.09 (mean ± SD) degrees and 77.69 ± 5.22 (mean ± SD) degrees, respectively. $P < 0.001$ (n = 8 for each group).

Supplementary Table S1. Cell wall acidification of wild type (Col-0) and *csi1-3* mutant

Sample	pH of medium (0 min)	pH of medium (150 min)
WT	6.05 ± 0.02	5.88 ± 0.08^{ns}
csi1-3	6.05 ± 0.02	5.87 ± 0.07^{ns}

Thirty four-day-old intact *Arabidopsis* etiolated seedlings of each genotype were incubated in depletion medium supplemented with 1 μM IAA. The pH of incubation medium was measured, from which cell wall acidification can be inferred. Data are mean ± SEM from three biological replicates. ^{ns} indicates statistically not significant. Statistical analysis was performed by two-tailed Student's *t*-test.

Supplementary Table S2. Dry mass per unit length measurement of wild type (Col-0), *csi1-3* and *jia1-1* mutants

Sample	Dry mass per unit length (µg/mm)
WT	1.75 ± 0.42
csi1-3	5.26 ± 2.11 ^{ns}
jia1-1	1.47 ± 0.18 ^{ns}

A group of 15 *Arabidopsis* hypocotyl segments from 3-day-old etiolated seedlings were used in each replicate for each genotype. The lengths of hypocotyl segments were recorded by individual groups and dry mass were measured after freeze-drying. Data are mean ± SEM from three replicates. ^{ns} indicates statistically not significant. Statistical analysis was performed by two-tailed Student's *t*-test.
